# Course-specific performance and mentor characteristics in an undergraduate tutorial system in medical education

**DOI:** 10.3389/fmed.2026.1862688

**Published:** 2026-07-20

**Authors:** Jinting Wu, Shengchan Wang, Yanyan Deng, Ni You, Shuchun Zhang, Liangjuan Yu, Yuting Xiong, Lijuan Wu, Hongxiang Zhang, Dasheng Lu

**Affiliations:** 1 Department of Psychology, The Second Affiliated Hospital of Wannan Medical University, Wuhu, Anhui, China; 2 Department of Health Management, Suzhou Vocational Health College, Suzhou, Jiangsu, China; 3 Department of Cardiology, The Second Affiliated Hospital of Wannan Medical University, Wuhu, Anhui, China; 4 Department of Gastroenterology, The Second Affiliated Hospital of Wannan Medical University, Wuhu, Anhui, China; 5 Department of Education, The Second Affiliated Hospital of Wannan Medical University, Wuhu, Anhui, China; 6 Human Resources Department, The Second Affiliated Hospital of Wannan Medical University, Wuhu, Anhui, China; 7 Outpatient Department, The Second Affiliated Hospital of Wannan Medical University, Wuhu, Anhui, China; 8 Department of ENT, The First Affiliated Hospital of Wannan Medical University, Wuhu, Anhui, China

**Keywords:** medical education, mentor characteristics, mentorship, theoretical performance, undergraduate tutorial system

## Abstract

**Background:**

Mentorship programs are widely used in medical education, but evidence linking specific mentor characteristics to students’ course-specific theoretical performance remains limited. This study examined associations between mentor characteristics and theoretical examination outcomes within an undergraduate tutorial system (UTS).

**Methods:**

This retrospective cohort study included 94 fourth-year medical students who participated in a structured UTS (each with one faculty mentor) and 136 non-UTS students for contextual comparison. Primary outcomes were final examination passing (≥60) and high achievement (≥80) in Internal Medicine and Surgery. Mentor characteristics included gender, age, professional title, academic degree, student-to-mentor ratio, and clinical specialty–course matching. Generalized Estimating Equations (GEE) accounted for clustering of students within mentors.

**Results:**

UTS students had higher mean theoretical scores than non-UTS students (71.1 ± 12.0 vs. 64.5 ± 9.4). Students performed better in Surgery than in Internal Medicine (passing rate: 86.2% vs. 71.3%, *p* < 0.001; high-achiever rate: 36.2% vs. 20.2%, *p* = 0.002). In multivariable GEE, having a mentor with a senior professional title was independently associated with higher odds of passing Internal Medicine (adjusted OR = 2.89, 95% CI: 1.05–7.96, *p* = 0.04). No other mentor characteristic showed a statistically significant association in multivariable analyses. In univariate analyses, younger mentor age was associated with high achievement in Internal Medicine (OR per year = 0.92, 95% CI: 0.85–0.99, *p* = 0.03).

**Conclusion:**

In this UTS program, a senior mentor title was independently associated with passing the Internal Medicine examination. Exploratory findings regarding mentor age require confirmation in larger, prospective studies. Course-specific differences in student performance were pronounced, suggesting that the impact of formal mentorship on theoretical learning is context-dependent.

## Introduction

In the field of medical education, the Faculty Mentoring Program, as a crucial educational model aimed at reducing anonymity between teachers and students and providing personalized guidance and support, is increasingly valued (1). Traditionally, medical education faces the challenge of “depersonalization” as it expands in scale, where students may interact with hundreds of teachers throughout their studies but lack sustained, in-depth mentorship relationships (2). To address this challenge, many medical schools have introduced structured mentorship programs with core objectives including: offering early professional exposure and clinical skill training for medical students (3); providing continuous support in coursework, career planning, and dissertation writing (1); and fostering the formation of professional identity through stable teacher-student relationships (4). Research indicates that effective mentorship not only enhances students’ understanding and confidence in specific specialties (such as otolaryngology) (3) but also significantly improves their overall satisfaction with the school’s career planning services (5). Additionally, longitudinal mentorship relationships are recognized as one of the effective strategies to address the disconnection between teacher-student, peer-peer, and doctor-patient relationships during clinical internships (6).

Although the benefits of mentorship in providing psychological support, career guidance, and clinical skill development have been widely observed (3, 7, 8), existing research primarily focuses on its impact on clinical competence, career interest selection, or research output (3, 9, 10). However, empirical studies on how mentorship affects the academic performance of medical undergraduates in core theoretical courses remain scarce. Mastery of theoretical knowledge is fundamental to medical education, and whether and how mentorship translates into more effective theoretical learning strategies and outcomes for students remains a key question yet to be fully explored.

Further analysis suggests that the individual characteristics of mentors may constitute significant potential factors influencing guidance effectiveness and student academic performance. Evidence indicates younger faculty and those from top-tier institutions are more likely to be “accessible mentors” (11). This implies that a mentor’s age, academic standing, or the prestige of their affiliated institution may affect the quality and frequency of teacher-student interactions. Meanwhile, to foster in-depth mentorship rooted in shared identity and values, new mentorship platforms are increasingly emphasizing the display of mentors’ professional and personal (identity) attributes (4). This indirectly suggests that features such as gender and personal background may play a role in establishing effective mentoring relationships. Additionally, a mentor’s title and degree are typically linked to their academic experience, resource networks, and teaching commitment, all of which may indirectly influence their ability to guide students in addressing theoretical challenges. Another critical structural factor is the student-to-mentor ratio (i.e., the number of students a mentor oversees), which directly impacts a mentor’s capacity to provide sufficient time and personalized attention to each student. Although a successful otolaryngology mentorship program adopted an approximate 1:2 ratio (3), the specific effects of varying ratios on theoretical performance remain unclear.

A recent scoping review of mentorship confirmed that while mentorship is increasingly recognized as foundational to clinical competency, scholarly engagement, and professional identity formation, existing research remains inconsistently structured, under-theorized, and variably evaluated, with few studies examining how specific mentor characteristics influence measurable learner outcomes (12). Therefore, the primary aim of this study was to explore associations between mentor characteristics (gender, age, professional title, degree, student-to-mentor ratio, and specialty matching) and medical students’ theoretical examination performance within a structured undergraduate tutorial system (UTS). As a secondary aim, we compared UTS participants with a non-UTS group on Internal Medicine scores, acknowledging the non-randomized design.

## Methods

### Study design and participants

This retrospective cohort study was approved by the Institutional Review Board. A total of 230 fourth-year medical students from a teaching hospital in China were enrolled: 94 students participated in the Undergraduate Tutorial System (UTS) and 136 did not (non-UTS). The non-UTS group was included only for descriptive comparison of scores. All UTS students were paired with one faculty mentor through bidirectional selection. The UTS program involved regular clinical teaching rounds, small-group lectures, skills training, and academic support. The system was overseen by the hospital’s Undergraduate Tutorial System Leading Group, which established a standardized framework to define tutor responsibilities and ensure consistent guidance quality across all participants. Under this framework, tutors were required to engage in regular interactive clinical guidance with students throughout the academic year, including teaching ward rounds, small-group lectures, clinical skills training, outpatient and surgical observation, and daily clinical teaching rounds, to maintain sustained student-tutor interaction. Tutors were also expected to provide targeted academic support, with their performance linked to students’ core course outcomes to incentivize proactive assistance with learning. Additionally, tutors were encouraged to foster students’ research and innovation capabilities by guiding them in academic paper publication, disciplinary competitions, and patent application activities. The framework also recognized tutors’ contributions to students’ long-term development, such as supporting postgraduate entrance examination preparation and admission. All participating tutors met the baseline requirements of the framework, ensuring the standardization and consistency of the UTS intervention across the study cohort. Students were divided into two groups based on their enrollment in the UTS: the UTS group and the non-UTS group. Participation in the UTS was voluntary, and mentoring pairs were formed through a bidirectional selection process based on mutual interest between students and faculty mentors, a method aligned with practices described in other mentoring programs.

### Data collection and outcomes

For UTS students, final examination scores in Internal Medicine and Surgery were collected. Mentor characteristics (gender, age, professional title [senior vs. non-senior], highest degree [primary vs. advanced], student-to-mentor ratio, and clinical specialty) were recorded. For each course, we defined specialty matching as the mentor’s specialty being the same as the course (e.g., internist for Internal Medicine; surgeon for Surgery). Theoretical course was defined as examinations assessing declarative and conceptual knowledge of disease mechanisms, diagnosis, and treatment principles, in contrast to clinical skills assessments. Both Internal Medicine and Surgery final examinations were written, knowledge-based tests focusing on factual recall and basic clinical reasoning, without hands-on skills components.

### Statistical analysis

Descriptive statistics are presented as mean ± standard deviation for continuous variables and as frequency (percentage) for categorical variables. Initial comparisons between the UTS and non-UTS groups were conducted using independent samples *t*-tests. To compare performance between Internal Medicine and Surgery among UTS students, paired t-test and McNemar’s test were used for continuous scores and binary outcomes, respectively.

To account for potential correlation among students supervised by the same mentor (clustering effect), we used Generalized Estimating Equations (GEE) with an exchangeable working correlation matrix for all regression analyses involving mentor characteristics and student binary outcomes (passing ≥60 points vs. failing; high achievement ≥80 points vs. not). This approach of categorizing outcomes is common in educational research (13). Separate GEE models were fitted for Internal Medicine and Surgery.

For each binary outcome, we first performed univariate GEE to examine the association of each mentor characteristic individually. Mentor characteristics included: age (continuous, years), gender (reference: female), professional title (reference: non-senior, i.e., junior/middle), highest degree (reference: primary degree, i.e., Bachelor’s), student-to-mentor ratio (continuous, as the number of students per mentor), and clinical specialty-course matching (reference: mismatched). Subsequently, a multivariable GEE model was built for Internal Medicine passing, including all mentor characteristics that showed a univariate association with *p* < 0.10. All tests were two-sided, and *p* < 0.05 was considered statistically significant. All statistical analyses were performed using Python 3.9. Core packages including numpy, pandas, scipy and statsmodels were adopted for numerical computation, data processing, bivariate statistical tests and generalized estimating GEE modeling.

## Results

### Mentor characteristics

A total of 230 fourth-year medical students were included in this retrospective cohort study, of whom 94 (40.9%) were in the UTS group and 136 (59.1%) in the non-UTS group. The mean theoretical examination score was significantly higher in the UTS group than in the non-UTS group (71.1 ± 12.0 vs. 64.5 ± 9.4). However, due to the lack of baseline academic data and non-randomized assignment, this comparison is descriptive only and should not be interpreted causally. As shown in Table 1, the 34 mentors had a mean age of 39.7 ± 5.8 years, 64.7% of mentors were male, 44.1% held a primary (Bachelor’s) degree as their highest degree, and 41.2% held a senior professional title. The mean teaching experience of mentors was 11.3 ± 6.8 years, and the average student-to-mentor ratio was 2.8 ± 0.5.

**Table 1:** Demographic characteristics of mentors in the undergraduate tutorial system.

Mentor characteristic	Value
Age (years)	39.7 ± 5.8
Gender (male, %)	22 (64.7)
Degree (primary, %)	15 (44.1)
Teaching experience (years)	11.3 ± 6.8
Title (senior professional title, %)	14 (41.2)
Student-mentor Ratio	2.8 ± 0.5

Mentor characteristics are reported for the UTS group only, as non-UTS students did not participate in the tutorial system and had no assigned mentors.

### Comparison between internal medicine and surgery (UTS group)

Among the 94 UTS students, performance was significantly better in Surgery than in Internal Medicine. The mean score was higher for Surgery (74.2 ± 12.9) than for Internal Medicine (68.1 ± 12.8); paired t-test gave a mean difference of 6.1 points (95% CI: 4.4–7.8, *p* < 0.001).

The passing rate (≥60) was 86.2% (81/94) for Surgery and 71.3% (67/94) for Internal Medicine. McNemar’s test showed statistical significance (χ^2^ = 14, *p* < 0.001). The high-achiever rate (≥80) was 36.2% (34/94) for Surgery and 20.2% (19/94) for Internal Medicine. Significant difference was detected (χ^2^ = 9.78, *p* = 0.002).

### Mentor characteristics and passing the examination (univariate GEE)

Table 2 presents univariate GEE results for passing (≥60) in Internal Medicine and Surgery, with ORs and 95% CIs adjusted for within-mentor clustering. For Internal Medicine, having a mentor with a senior professional title was significantly associated with higher odds of passing (OR = 3.40, 95% CI: 1.35–8.55, *p* = 0.01). None of the other mentor characteristics (age, gender, degree, teaching experience, student-to-mentor ratio, or specialty matching) reached statistical significance. For Surgery, a higher student-to-mentor ratio was associated with increased odds of passing (OR = 2.85, 95% CI: 1.03–7.85, *p* = 0.04). No other mentor characteristic was significantly associated with passing Surgery (all *p* > 0.05). Given the absence of other significant predictors and the small sample size, we did not perform multivariable adjustment for this isolated finding. This result should be considered exploratory and interpreted with caution.

**Table 2:** Course-specific comparison of mentor characteristics between passing and failing groups in univariable analysis.

Mentor characteristic	OR	*P*	95% CI
Internal medicine			
Age	1.07	0.09	0.99, 1.15
Gender	0.50	0.22	0.16, 1.52
Degree	0.43	0.09	0.17, 1.12
Teaching experience	1.03	0.33	0.97, 1.11
Title	3.40	0.01	1.35, 8.55
Student-mentor Ratio	1.49	0.46	0.52, 4.24
Clinical specialty	1.55	0.38	0.58, 4.11
Surgery			
Age	1.01	0.80	0.91, 1.13
Gender	0.67	0.59	0.16, 2.84
Degree	0.51	0.29	0.14, 1.79
Teaching experience	1.04	0.33	0.96, 1.13
Title	2.72	0.14	0.73, 10.16
Student-mentor Ratio	2.20	0.21	0.65, 7.45
Clinical specialty	0.59	0.40	0.14, 2.22

Reference categories: gender = female; professional title = non-senior; degree = primary; specialty matching = mismatched.

### Multivariable GEE for internal medicine passing

Because only senior title showed a significant univariate association with passing Internal Medicine, and age as well as degree is a potential confounder, we fitted a multivariable GEE model including both age and professional title. As shown in Table 3, senior title remained independently associated with higher odds of passing (adjusted OR = 2.89, 95% CI: 1.05–7.96, *p* =  0.04), while age (adjusted OR = 1.00, 95% CI: 0.95–1.06, *p* = 0.94) and degree (adjusted OR = 0.58, 95% CI: 0.23–1.47, *p* = 0.25) were not significant.

**Table 3:** Course-specific comparison of mentor characteristics between passing and failing groups in multivariable analysis.

Internal medicine	OR	*P*	95% CI
Age	1.00	0.94	0.95, 1.06
Title	2.89	0.04	1.05, 7.96
Degree	0.58	0.25	0.23, 1.47

### Mentor characteristics and high achievement (univariate GEE)

Table 4 shows univariate GEE results for high achievement (≥80). For Internal Medicine, a younger mentor age was associated with higher odds of high achievement (OR per one-year increase = 0.91, 95% CI: 0.84–0.99, *p* = 0.03). No other characteristic was significant. For Surgery, none of the mentor characteristics reached statistical significance (all *p* > 0.05).

**Table 4:** Course-specific comparison of mentor characteristics between the high- achiever group and the non-high- achiever group in univariable analysis.

Mentor characteristic	OR	*P*	95% CI
Internal medicine			
Age	0.91	0.03	0.84, 0.99
Gender	0.58	0.20	0.25, 1.35
Degree	1.08	0.88	0.42, 2.73
Teaching experience	0.96	0.33	0.88, 1.05
Title	0.80	0.65	0.31, 2.09
Student-mentor ratio	1.00	0.99	0.36, 2.74
Clinical specialty	1.03	0.96	0.39, 2.68
Surgery			
Age	0.95	0.27	0.86, 1.04
Gender	0.60	0.27	0.24, 1.48
Degree	1.41	0.46	0.56, 3.54
Teaching experience	0.95	0.27	0.86, 1.04
Title	0.99	0.99	0.42, 2.32
Student-mentor Ratio	1.97	0.17	0.75, 5.22
Clinical specialty	1.11	0.81	0.46, 2.67

## Discussion

**Figure 1:**
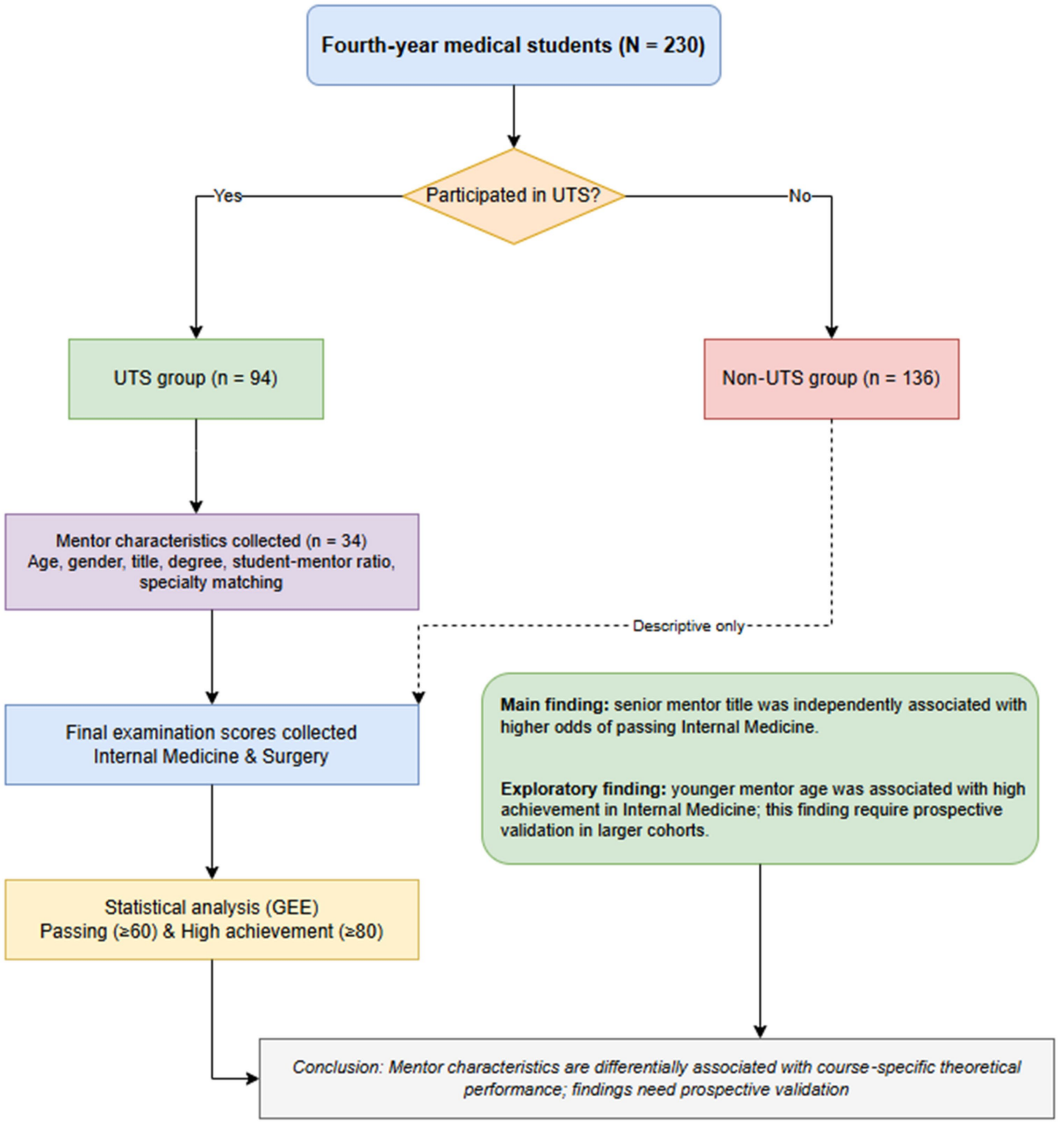
Flow chart and summary of the study.

This study examined associations between mentor characteristics and theoretical examination performance within an undergraduate tutorial system. UTS students scored higher on the examination than non-UTS students. This finding is consistent with existing literature on the benefits of mentorship. Mentorship helps reduce “anonymity” and “depersonalization” in large-scale medical education by providing personalized academic support and consistent teacher-student interaction (1, 2). Structured mentoring offers students early clinical exposure and skill development (3). It also strengthens students’ ability to integrate and apply knowledge through ongoing feedback and guidance (2, 5). We found students performed significantly better in Surgery than in Internal Medicine, both in passing rates and high-achiever rates. In terms of mentor characteristics, a senior professional title was independently associated with higher odds of passing Internal Medicine. Additionally, younger mentor age was associated with higher odds of achieving top scores (≥80) in Internal Medicine (Figure 1).

### Course-specific differences

The superiority of Surgery over Internal Medicine was robust across two thresholds and confirmed by paired analyses that accounted for within-student correlation. Several explanations may account for this disparity. Surgery examinations often test factual knowledge, anatomical relationships, and procedural steps, which may be more amenable to memorization. In contrast, Internal Medicine requires integrated clinical reasoning, differential diagnosis, and management of complex, multifaceted cases, which is typically more challenging for students at this stage. Previous research on an otolaryngology mentorship program showed that early clinical and operating room exposure significantly enhanced participating students’ confidence in clinical performance and knowledge of anatomy compared to non-participants (3), which might explain why Surgery appears to benefit more from the tutorial system. Differences in teaching methods, student interest, or examination difficulty and grading standards between departments may also contribute. Regardless of the mechanism, this finding has practical implications. Educators should examine whether Internal Medicine instruction or assessment could be modified to improve student outcomes, or whether such differences are expected and acceptable. Importantly, the higher baseline performance in Surgery might also influence the interpretation of mentor effects: the near-ceiling passing rate (86.2%) left little room for mentorship to show an association, whereas the lower pass rate in Internal Medicine (71.3%) provided greater variability, potentially allowing the effect of senior title to become detectable.

### Senior title and passing internal medicine

The robust association between senior mentor title and passing Internal Medicine suggests that academic seniority may help students avoid failure in more cognitively demanding subjects. Senior faculty likely possess refined pedagogical skills and a deep understanding of curricular fundamentals. Previous research has shown that presenting the ‘how and why’ of disease manifestations can significantly enhance students’ learning outcomes (14). This finding echoes observations that a mentor’s academic standing can influence their accessibility (11). Interestingly, senior title was not associated with high achievement, implying that excelling at a high level may require additional factors such as student intrinsic motivation, study habits, or mentor-mentee interaction quality.

### Younger mentor age and high achievement in internal medicine

We found that younger mentor age was associated with higher odds of achieving ≥80 in Internal Medicine. This aligns with previous research showing that younger faculty are more accessible (11). However, given the narrow age range of mentors and the modest effect size, this finding should be interpreted with caution. Besides, the association was only present for high achievement (not for passing) and only in Internal Medicine, suggesting that age effects might be domain- and outcome-specific. Furthermore, due to the limited sample size, replication is needed in future studies.

### Lack of associations for other mentor characteristics

The associations between mentor personal characteristics and mentee academic performance remain controversial. A recent qualitative study showed that female mentors in Surgery provide valuable networking, career guidance, and unique insights (15). However, mentor gender was not associated with students’ examination performance at a statistically significant level in our cohort. It is consistent with the view that the success of mentoring hinges on the quality of the mentoring behaviors—such as mentor guidance ability, relationship-building skills and communication frequency—rather than on inherent characteristics like age, gender, or background (8, 16, 17).

### Limitations and future directions

Several limitations must be acknowledged. First, this was a single-center retrospective study, limiting generalizability. Second, we lacked pre-intervention academic data (e.g., prior GPA), which precludes adjustment for selection bias in the UTS vs. non-UTS comparison. However, previous study suggests that students’ demographic characteristics are generally not predictive of outcomes, whereas factors such as mentor expertise and regular involvement are consistent predictors of academic and non-academic benefit (8). Future studies can combine questionnaires or interviews to explore subjective and process-related variables, such as specific mentoring processes, interaction frequency, guidance content, and teacher-student relationship quality. Finally, the definition of “theoretical” was based on written examination format; whether these results apply to clinical performance assessments requires further investigation.

## Conclusion

In this single-center UTS program, students performed substantially better in Surgery than in Internal Medicine. Having a senior mentor was independently associated with passing Internal Medicine. Younger mentor age correlated with high achievement in Internal Medicine, but these findings require replication and cautious interpretation. No other mentor characteristics showed consistent associations. Future prospective, multi-center studies with larger samples, baseline ability measures, and detailed process data are needed to clarify the conditions under which mentor characteristics influence theoretical learning.

## Data Availability

The original contributions presented in the study are included in the article/supplementary material, further inquiries can be directed to the corresponding authors.
